# Recoverable Earth: a twenty-first century environmental narrative

**DOI:** 10.1007/s13280-018-1065-4

**Published:** 2018-06-09

**Authors:** Paul Jepson

**Affiliations:** School of Geography and the Environment, Dyson Perrins Building, South Parks Road, Oxford, OX1 3QY UK

**Keywords:** Conservation policy, Environmental narratives, Landscape restoration, Policy narratives, Rewilding

## Abstract

Rewilding may signify the emergence of a new environmental narrative. Discussion of underlying policy narratives is important because they shape understandings of the state of world and how society should act. I summarise the origins of twentieth century environmental narratives and argue that their influence derives from components telling of the dire state of nature, the catastrophic consequences of this and the need for competent authorities to act to govern the perpetrators of harm. Reflecting on my engagements with rewilding science and practice, I posit that stories of rewilding are adopting a quite different narrative structure: one that involves components telling of feelings of despondency and processes of awakening, action, and reassessment leading to the recovery of natural and social well-being. These components align with the narrative structure of accounts of mental health. I label this emerging narrative ‘Recoverable Earth’ and suggest that it signifies action by grassroot conservationists to reassert their ability to lead change locally and produce better outcomes for nature and society.

## Introduction

Here I posit the emergence of a new European environmental narrative that I label ‘Recoverable Earth’. I argue that this new narrative is gaining coherence and popular meaning through pioneer projects and rhetoric associated with the European Rewilding Network. Further, I contend that, in structure, ethos, worldview and agenda, it is distinct from the powerful narratives that shaped twentieth century institutions of conservation action and environmental policy. To position and contrast this new narrative, I start by briefly describing the origins, architecture and power of two twentieth century environmental narratives. I then present accounts of engagements with rewilding practitioners that suggest similarities between their articulation of rewilding visions and the more reflexive narrative architecture of stories of mental health recovery. These foreground independence of action and a willingness to reassess beliefs and expectations and shape new futures. The Recoverable Earth narrative I outline is appealing, empowering and confident and places the restoration of ecological systems at the centre of societal change.

An appreciation of underlying environmental narratives is important. This is because narratives are a guide to sense making in a complex and uncertain world. Through assigning structure and meaning to entities and processes, they give social movements and associated advocacy coalitions legitimacy and purpose (Sabatier 1993). Environmental narratives frame problems and issues in ways that are meaningful and compelling for publics and policy makers. They are always political, overtly or otherwise. This is because narratives create an ‘architecture’ for the telling of normative stories about the state of the world, the consequences of this for humanity, and what needs to be done.

## The rise and structure of twentieth century environmental narratives

An in-depth review of twentieth century environmental narratives is beyond the scope of this article. Instead, I would like to characterise two narratives of environmental concern and policy that I think are broadly recognisable to all. The origins of environmentalism lie with a group of social movements that emerged during the late nineteenth/early twentieth century in the colonies and cities of Western Europe and North America (Jepson 2017). Prominent citizens mobilised to avoid the extinction of species, the devastation of wildlife populations, rapid depletion of natural resources, the destruction of nature monuments and urban green space, and (in the USA) the protection of wilderness. Their appeals to action foregrounded two narratives: one aspirational, that linked nature protection with the realisation of civilised values (Jepson and Whittaker 2002) and a second, risk-based narrative that emphasised the threats to social, economic and territorial instability arising from damage to watersheds, soil erosion and declines in strategic resources such as timber (Grove 1992).

My reading of the literature of these times suggests that whilst causality was a key component of these formative conservation narratives the attribution of blame was not a major constituent. The destruction of nature by market hunting, pioneer agriculture, the spread of diseases (notably rinderpest) and industrialisation were generally framed as unfortunate outcomes of the ‘march of civilisation’, which could be addressed through regulations to protect species, governing hunting and by creating sanctuaries, parks and resource reserves. US President Theodore Roosevelt’s 1909 value articulation that ‘*human conquest of nature carries with it a moral responsibility to ensure the survival of threatened life forms*’ (Hornaday 1914) expresses this worldview. This conservation value inspired the wildlife movement and the near universal adoption of policies to avoid species extinctions (Ladle and Jepson 2010): it accepts the reality of human exploitation and modification of nature and frames the act of saving and protecting nature as a moral cause. In narrative terms, it presents conservation as the extension of civilised values of compassion, stewardship and moral consideration to the non-human world: an act that would ennoble humanity and, in the wake of Darwinism, help humans reclaim their special identity (for context see Jepson and Whittaker 2002).

Post WWII, a new environmental narrative took root in the US that more explicitly foregrounded environmental limits. Popular books, such as Fairfield Osborn’s *Our Plundered Planet* (1948), William Vogt’s (1948) “*Road to Survival*” and later Rachel Carson’s ‘*Silent Spring*’ (1962) led to a more structured, urgent and compelling narrative. In contrast to earlier conservation narratives that told of vanishing species and damage to places largely disconnected from western publics, these new stories told of apocalyptic scenarios for humanity caused by human greed, profligacy, ignorance, fecundity and poor stewardship. They had impact because the physical manifestations of the arguments, such as water pollution, smog, litter and the collapse for predatory bird populations, were becoming visible to all, urban and rural alike. This was particularly so in the US and Western Europe where a new environmental consciousness manifested in the 1970 Earth Day: environmentalism was taken up by a young, well-educated-, middle class generation with the activist confidence of 1960s counter culture. They populated the narrative with villains—polluting industries, agro-chemical companies, Russian whaling fleets, complacent government agencies and so forth—who could become the target of campaigns. Often these campaigns involved media-friendly direct actions involving heroic eco-warriors confronting the perpetrators of environmental harm, which evoked deeper cultural narratives e.g., Greenpeace’s David vs Goliath anti-whaling campaigns of the 1970s (Day 1987).

**Fig. 1:**
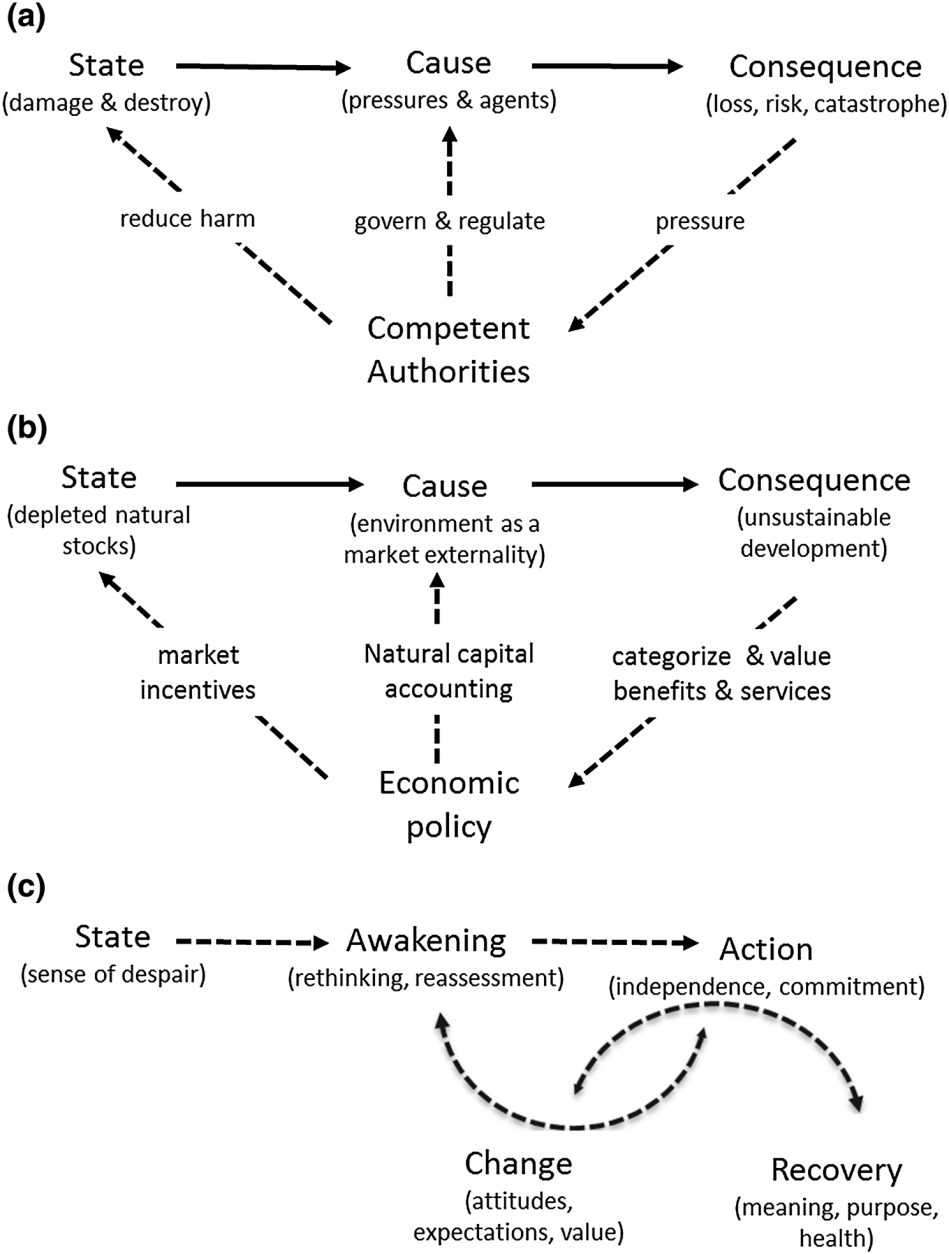
Simplified structure of environmental narratives. **a** Finite Earth narrative: 1970s onwards. **b** Resource Earth narrative: 1990s onwards. **c** Recoverable Earth narrative: emerging

This is what I label the “Finite Earth’ narrative (Fig. 1a). It conforms to (and may have informed) a common structure for policy narratives: stories of worrying change and transformation, populated with villainous, heroic and innocent characters (human and non-human), and appeals to decision-making elites to act morally and as a force for good (Stone 1989). Accounts of the rise of modern environmentalism (e.g., Whitaker 1976) point to a significant shift in the worldview underpinning conservation policy and action during this period: earlier confidence in the capacity of groups to protect, manage and reshape nature was replaced by a deep sense of despair concerning humanity’s ‘wounding ways’. A logic emerged that humanity could no longer be trusted to act responsibly in relation to the environment and areas should, therefore, be set aside where nature could take its course. This logic had resonance with the US wilderness movement and among ecologists whose discipline was gaining in policy influence. It introduced a preservationist worldview to the conservation narrative, which remains influential today, namely that nature conservation should be an end in itself (see, e.g., Noss 1990).

A policy narrative has greater impact when it is strategically constructed by coalitions of actors who adopt similar problem perceptions and causal assumptions and agree what constitute viable actions and solutions within particular policy and political contexts (Jones and McBeth 2010; Shanahan et al. 2011). The 1980 World Conservation Strategy (IUCN/UNEP/WWF 1980) signified the beginning of efforts to strategically align the environmental/conservation narrative with the higher level narrative of sustainable development (Nicholson 1986). This process gained momentum when conservation biologists coined the term biodiversity in the late 1980s (Wilson 1988) and warned of a sixth extinction crisis and the risk of collapse of the ecosystem functions upon which life depends (Ceballos et al. 2015). This articulation of the state–cause–consequences narrative logic was formalised in the text of the 1992 Convention on Biological Diversity. International policy actors aligned biodiversity with developmental agendas of poverty alleviation, rural livelihoods, and social justice. They did so by framing biodiversity as a new form of natural resource that could be systematically surveyed, prospected and developed (Haila and Kouki 1994) and that represented the fundamental units of nature producing the ecosystem services required for sustainable development. In the biodiversity narrative, inter-governmental and government agencies retained their role as the authorities with the responsibility and competencies to address the problems, which they perused through Integrated Conservation and Development projects, delivered in conjunction with NGOs and consultancy partners (Wells et al. 1998).

This process of ‘mainstreaming’ conservation and environmental narratives with wider development policy continued with Costanza et al.’s seminal (1997) paper *“The value of the world’s ecosystem services and natural capital”*. They argued that because ecosystem services contribute to human welfare they represent part of the total economic value of the planet and estimated the value of these to be in the region of $16–54 trillion per year (compared to a Global GNP of ca. US $18 trillion per year). In this new framing, the state of the nature was considered worrying because it represents a loss of economic value, caused by environmental externalities (costs of environmental damage external to the function of markets) and leading to development that is unsustainable. In response, economists developed the concept of ‘natural capital stocks’ that can be valued (even if imperfectly), aggregated and accounted for in economic decision-making and performance reporting, and that enable the creation of markets to pay for the production of ecosystem services (Helm 2015).

In my view, these alignments created a new ‘Resource Earth’ narrative that initially adopted a similar structure to the earlier ‘Finite Earth’ narrative (Fig. 1a, b), but which differs in two important respects: (i) economic policy has replaced government authority as the entity with the power to avert the consequences of environmental damage and (ii) the dichotomy between characters of good and evil is weaker—perpetrators of environmental harm may lack the economic frameworks and incentive to do the right thing rather than being morally bad.

The last 20 years has seen an explosion of research on the linked concepts of ecosystem services and natural capital which have gained considerable traction in policy (Costanza et al. 2017). A fuller examination of this literature is likely to reveal a deferent and perhaps more complex structure to the Resource Earth narrative. For example, the narrative maybe moving towards a more systems architecture with narrative elements relating to stocks, drivers, dependencies, benefits/services and valuation.

## Rewilding and the formulation of a new conservation narrative

The idea of rewilding is the subject of a growing academic and popular discourse. This debates rewilding from the critical perspectives of a wide range of discursive fields and agendas. These range from conservation baselines and targets (e.g., Donlan et al. 2006) to environmental hermeneutics (e.g., Drenthen 2013) and restorative justice (e.g., Monbiot 2013). Such discourse is, however, poorly linked to practice (Svenning et al. 2016) and the voices of those initiating and leading rewilding projects are mostly absent. To paraphrase, Sandom et al. (2012) practical expressions of rewilding may best be understood as *spaces of innovation in conservation management, theory and philosophy characterized by a desire to restore ecosystem dynamics and functions at various scales often through the introduction of functional species’*.

My aim is to draw attention to the narrative structures associated with these practical spaces of conservation innovation rather than to engage in the wider discursive tussles. My focus is a mode of rewilding practice that is gaining momentum through the actions of Rewilding Europe (est. 2012). This has its origins in radical new policy visions for Dutch river management that emerged during the 1980s and was integrated into Dutch nature and landscape policy under the label ‘nature development’ (Van Den Belt 2004). It is guided by the principles of: (i) restoring natural processes and dynamics, (ii) taking inspiration from the past to shape new natures, (iii) creating self-sustaining ecosystems, and (iv) and working with restored forces of nature to reconnect nature conservation with modern society and economy (Jepson and Schepers 2016). In this version of rewilding, the ‘re’ prefix means again, not back.

The growing European rewilding network is telling fresh and compelling stories. Stories of restoring trophic interactions, food chains and river and grazing dynamics, of the return and recovery of mega-fauna, of re-finding the self and restoring trust in nature, society and economy. Such rewilding stories tell of the power of demonstration projects to develop novel solutions to environmental and social change. These are stories of hope, vision and ambition that inspire and empower (see, e.g., Rewilding Europe 2017).

These new conservation stories lack the narrative elements of the established environmental narrative. Whilst the poor state of nature is recognised the crucial ‘catastrophic consequences’ component is absent, as are the elements of blame and appeals to higher authorities (or economics) to regulate the perpetrators of environmental harm. Instead, rewilding stories foreground new ways of thinking and grounded adaptive action, intertwined with ideas of nature as a creative force and the prospect of a better future for all—people and nature (Fig. 1c).

In my view, there are strong similarities between the narrative elements of rewilding stories and those of mental health recovery as described by Ralph (2005) and Mancini and Rogers (2007). These authors present a narrative framework with five elements, namely: (i) accounts of despair, anguish and hopelessness, followed by (ii) an awaking phase involving accounts of hope of recovery, (iii) an action phase involving a commitment to wellness, and (iv) a process of reassessment (of feelings, roles, goals and expectations), leading to (v) the recovery of wellness. In stories of mental health recovery, process and independence of action and spirit are central to the narrative (cf. Anthony 1993) and the components are assembled in the form of reflexive progression, where, for instance, actions simultaneously interplay with awakening and the recovery of well-being.

When telling the story of their influential Knepp wildlands project, Sir Charles Burrell and Isabella Tree (co-founders of Rewilding Britain) adopt this narrative model (Tree 2017). They first tell of the struggle to farm their ancestral estate and the ‘*cycle of drudgery*’ that efforts to intensify agriculture brought to their land and lives. Then, they describe how a project to restore parkland around the house produced a ‘*psychological breakthrough*’ (p. 206) that opened their eyes to fresh possibilities. This led them to engage with Vera’s ground-breaking ideas on grazing and wood-pasture dynamics (Vera 2000) and a commitment to experiment with a new model of farming involving free roaming herbivores, the removal of fencing and the relaxation of land management. They then talk about the emergent properties of their ‘process-led’ and ‘open-ended’ experiment in farming, foregrounding the recovery of wildlife and ecosystem services and the recovery of their work and life quality (Burrell 2017).

There is some evidence that the ‘doom and gloom’ narratives may promote anxiety and a sense of futility thereby contributing to depression (Kelsey 2014; Pihkala 2017). I experienced this during a phase of my life when I worked on the Sumatran frontier. I witnessed and came to understand the ungovernable forces of forest destruction (Jepson et al. 2001) and with this internalised a sense of deep despondency concerning the future of the conservation values that defined my identity. This was a dark period for me when the wailing crescendos of Radiohead’s OK Computer album became the sound track of my life.

My awakening phase arose around 2006 from two interlinked sources. My M.Sc. Biodiversity students made it clear that they did not want to be taught a science that positioned them as cataloguers of an impending crisis: instead, they were seeking the theories, knowledge and insight that would empower them to shape a better future. This prompted me to lead a series of field trips to the Netherlands to visit rewilding projects and engage with the thinking of progressive Dutch ecologists.

Frans Vera’s story of the Oostvaardersplassen (OVP), a high profile public experiment in rewilding (Lorimer and Driessen 2014) awakened me to new and inspiring possibilities in conservation practice. In his OVP lecture (which I have heard several times), Vera (2017) adopts a structure that expresses the ‘Recoverable Earth’ narrative in a powerful and challenging way. He opens by comparing the European landscape to a Turkish carpet that has been cut into pieces and lost its beauty and function. Next, he describes his observations of grazing geese and the spontaneous development of vegetation on the OVP polder and how these observations awakened him to the role of grazers in driving vegetation dynamics and to the capacity of nature to recover unaided. He continues with the action component, telling of how he, together with other radical young ecologists working within the State nature agency convinced the authorities to allow them to establish a large herbivore guild on the OVP [comprising of Heck cattle, Konick horses and red deer (*Cervus elaphus*)], to test and develop natural grazing approaches to reserve management. Vera then goes on to describe how these experiments in management caused him to reassess established Clementsian notions of vegetation succession and inspired him to develop his theory of cyclic vegetation turnover (Vera 2000). He concludes his OVP lecture with accounts of the recovery of ecological dynamics, of flourishing bird populations and the return of species long extinct in the Netherlands such as White-tailed eagle (*Haliaeetus albicilla*).

Among my colleagues in Oxford, this ‘Vera unsettling’ prompted a collective, multi-disciplinary and on-going reassessment of the scientific tenets informing conservation policy. The insights of long-term ecology colleagues were particularly influential. They ‘awakened’ us to the reality that nature existed in multiple past states (or natural archetypes) and that although these may have experienced long periods of stability they are always in transition. In the context of climate change, this prompted the realisation that there is no way back and ecological restoration can only take insight and inspiration from the past to shape future natures (cf. Marris 2013). Linked to this was the realisation that the well-known ‘shifting baseline syndrome’ (Pauly 1995) in fisheries management applied to nature conservation, and that over time we have come to internalize ecological impoverishment in our culture, policy and institutions. This reassessment, and the recognition that the natures we choose to conserve are largely a cultural decision was empowering because it opened the prospect of creating new natural assets alongside the protection of the old. As a result, we now teach a more hopeful, inter-disciplinary and forward-looking biodiversity science, summarised as ‘protect the best, restore the rest’. Together with our students, we have recovered a sense of enthusiasm, excitement and purpose in our science and teaching.

Beyond structure, these European rewilding stories express a new philosophy of conservation action. Wouter Helmer, a founding member of Rewilding Europe, is a leading contributor to this new philosophy whose thinking is informed by his experiences leading hugely innovative, risky and successful rewilding projects at Gelderse Poort and Kempen-Broek in the Netherlands. In contrast to the campaigning ‘what needs to be done’ ethos of twentieth century environmentalism, Helmer’s approach is based on showing what can be done by allowing vision and practice to interact. Central to his rewilding action philosophy is the idea that change and uncertainty are at the core of all things and this reality can be embraced to shape new and better realities if we free our imaginations, take opportunities when they arise and re-find trust in people, economy and society. In this spirt, rewilding projects are initiated with invitations to collectively envision future landscapes and embark on uncertain, yet potentially rewarding journeys of change. In these unfolding stories of the future animals such as wilded horses & cattle, bison (*Bison bonasus*), deer, beaver (*Castor fiber*) and lynx (*Lynx lynx*) assume character roles because they generate interest and engagement among diverse publics. This philosophy of action is producing novel cultural landscapes with more room for natural dynamics and processes. These in turn are generating multiple forms of value for nature, people and society (cf. Jepson et al. 2017) and new forms of ecological knowledge that are generating nature-based solutions to societal problems (cf. Nesshöver et al. 2017), including climate change adaptation and rural depopulation. (Helmer 2015 and pers. comm.).

## Discussion

I have argued that pioneer rewilding projects, interacting with interdisciplinary conservation science are giving form to a new environmental narrative. At present these narratives are emerging at the micro- and meso-level (sensu Shanahan et al. 2011): they are present in the stories of grounded conservation networks and the communications of Rewilding Europe. I hope this perspective will prompt others to appraise the validity of my proposition that a new narrative is emerging and that in structure and ethos it is fundamentally different to those that were institutionalised last century. It is also an invitation to adopt, promote and shape this new narrative such that it assumes an institutional and cultural presence.

For me, the Recoverable Earth narrative embodies an underlying worldview identifiable with pragmatic realism (see El-Hani and Pihlström 2002). This views nature and society as intertwined: natural entities exist independent of human consciousness but how we conceptualise and interact with them structures their identities, abundance, distribution and associations. It understands the degraded state of nature as the outcome of complex interactions between nature, culture, politics and economy over the long term. Given this, there is little value in feeling guilt and attributing blame: we are where we are and there is no way back, yet pragmatically, we can engage with the forces of nature and complex human societies to shape the future.

The twentieth century environmental narratives are consistent with the techniques of anxiety marketing (Sachs 2012): through appeals to reduce harm and avoid the risk of impending catastrophe they mobilise governments and publics to act to protect and conserve the environment. These anxiety-based narratives have gained such cultural prominence, power and stability that they have become paradigmatic. However, they have also come to hold a ‘lock’ on the imaginations of decision-makers, scientists and activist publics (see McCarthy and Cramb 2009) and may be out of touch with the public mood and wider trends in society. The Recoverable Earth narrative offers a fresh and empowering environmental narrative that can complement and extend the established narratives. It offers citizens something new, hopeful, intriguing, purposeful and potentially rewarding, namely the invitation to participate in the unfolding of new stories about the relationship between nature and people. These stories are populated by animal characters and natural forces and involve stories of people and organisations coming together in a spirit of trust to imagine and work with natural dynamics to restore landscapes where nature and people flourish in a changing and uncertain future.

If, as I suggest this Recoverable Earth narrative is real, the question arises how will one interact with the Finite Earth and Resource Earth narratives. I am aware of concerns that a narrative which communicates the message that environmental damage is recoverable could be exploited by lobbies seeking to weaken environmental legislation. Rather than constituting a policy risk, my view is that the Recoverable Earth and Finite Earth narratives are complementary and, in interaction, could reinvigorate the conservation and environmental movements. The twentieth century narratives interacted with notions of the risk society (Beck 1992) where polities respond to manufactured risks (those produced by modernisation). This interaction has brought huge environmental benefits, but I suggest that it has also promoted a deep sense of despondency and maybe even learnt helplessness (Klein et al. 1976) among grassroot conservationists and citizens. Collectively, we feel increasing guilt at what we are collectively doing to nature yet seem beholden to centralised and self-interested polities to do something about it. The Recoverable Earth narrative offers a way out of this depressing situation by suggesting that rather than beating ourselves up for the harm we have inflicted on our planet, we can work with the forces of nature and within the constraints of society to recover the biosphere and through this find new meaning and purpose in our lives. From this perspective, rewilding can be viewed as actions of the grassroot conservation movement to recover from its malaise by reasserting an ability to lead change locally leading to better outcomes for nature and society.

There will always be a need for professional conservation lobbyists pressuring governments and corporations to adopt legal and policy frameworks to conserve nature and the environment. In spaces of high politics the *Finite Earth* and *Resource Earth* narratives are likely to remain effective. However, society is moving towards more distributed, networked and localised governance and organisational structures. To mobilise and empower these, the Recoverable Earth narrative may be just the ticket.
